# Ultrasound Induced Fluorescence of Nanoscale Liposome Contrast Agents

**DOI:** 10.1371/journal.pone.0159742

**Published:** 2016-07-28

**Authors:** Qimei Zhang, Stephen P. Morgan, Paul O’Shea, Melissa L. Mather

**Affiliations:** 1 Advanced Optics Group, Faculty of Engineering, University of Nottingham, Nottingham, United Kingdom; 2 Cell Biophysics Group, School of Life Sciences, University of Nottingham, Nottingham, United Kingdom; 3 Institute for Science and Technology in Medicine, Keele University, Stoke-on-Trent, United Kingdom; Oregon State University, UNITED STATES

## Abstract

A new imaging contrast agent is reported that provides an increased fluorescent signal upon application of ultrasound (US). Liposomes containing lipids labelled with pyrene were optically excited and the excimer fluorescence emission intensity was detected in the absence and presence of an ultrasound field using an acousto-fluorescence setup. The acousto-fluorescence dynamics of liposomes containing lipids with pyrene labelled on the fatty acid tail group (PyPC) and the head group (PyPE) were compared. An increase in excimer emission intensity following exposure to US was observed for both cases studied. The increased intensity and time constants were found to be different for the PyPC and PyPE systems, and dependent on the applied US pressure and exposure time. The greatest change in fluorescence intensity (130%) and smallest rise time constant (0.33 s) are achieved through the use of PyPC labelled liposomes. The mechanism underlying the observed increase of the excimer emission intensity in PyPC labelled liposomes is proposed to arise from the “wagging” of acyl chains which involves fast response and requires lower US pressure. This is accompanied by increased lipid lateral diffusivity at higher ultrasound pressures, a mechanism that is also active in the PyPE labelled liposomes.

## Introduction

Fluorescence imaging has become a cornerstone technology which enables direct visualization of the physiological processes in a living cell or tissue as well as extraction of unique functional information about biological systems and their microenvironment [1–2]. The application of fluorescence techniques to tissue imaging, however, is limited by the high optical scattering and absorption properties of tissue [3–6]. To improve the imaging capabilities of fluorescence techniques, methods based on models of photon transport in tissue combined with image reconstruction have been investigated including continuous-wave (CW) fluorescence diffuse tomography, time-domain fluorescence diffuse tomography and frequency-domain fluorescence diffuse tomography [7]. Whilst these techniques provide some improvement to image quality they require solution of an inverse problem, which has inherent assumptions about the nature of light propagation and can be computationally expensive.

Recently techniques based on ultrasound mediated fluorescence emission have been investigated to address the effects of light scattering and provide fluorescence imaging of tissue with spatial resolution of the order of a few millimeters [8–17]. In these approaches fluorescence emission is confined only from the regions of tissue exposed to ultrasound, which is the basis of improved spatial resolution. Enhancement of this effect is possible through the incorporation of fluorophores in gas filled microbubbles composed of lipid shells which act as contrast agents. Fluorophores of an identical type [18–20] or with unlike quenchers [21] are added at a sufficient concentration to cause fluorescence self-quenching (between the identical fluorophores) or fluorescence resonance energy transfer (FRET) (between the donors and quenchers) in the absence of US. With the application of US, volumetric oscillation of the microbubbles occurs changing the intermolecular distance of fluorophores located at the bubble surface and hence the extent of self-quenching or FRET. Significant enhancement of the ultrasound modulated fluorescent signal has been demonstrated [18–21]. Despite the above improvements, the use of microbubble contrast agents for tissue imaging is technically challenging due to their instability to multiple US pulses, short circulation time and heterogeneous size distributions. It is also intrinsically difficult to evenly distribute fluorophores on the lipid shell due to the formation of concentrated lipid islands, which compromises the achievable modulation of fluorescent emissions. Furthermore, for downstream in vivo applications the microscale size of bubbles restricts the use of these contrast agents to the circulatory system, since microbubbles stay within the vascular compartment and do not leak out into the extra-vascular space [22].

Here we present for the first time to our knowledge a new approach to enhancement of fluorescent signals by US based on the use of nanoscale excimer-emitting liposomes. Liposomes are spherical shaped structures composed of one or more bilayers of phospholipid molecules with a liquid core that can be produced with defined diameters ranging from tens of nanometers to several micrometers [23]. In comparison to microbubbles, liposomes are more stable and can be manufactured with tight size distributions. Because of their liquid core, liposomes will produce less perturbation of the US field as compared to microbubbles, which is beneficial for image resolution as the US field is more localized. Due to the nanoscale size they can pass through vascular walls to potentially overcome the blood brain barrier. They are also biodegradable, biocompatible and can be prepared with defined circulation lifetimes within the body [24–25].

**Fig 1:**
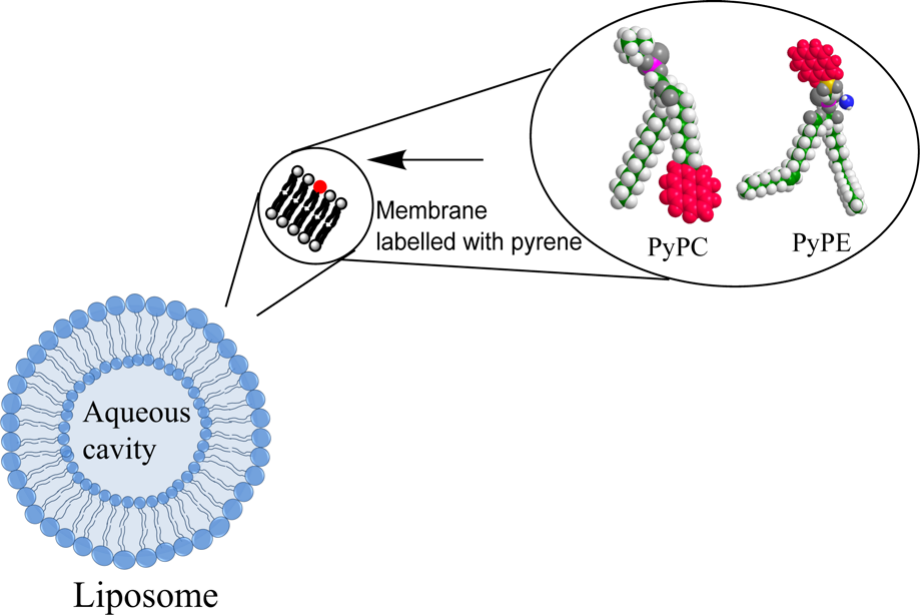
Schematic showing liposome containing pyrene labelled phospholipids. The expanded section depicts lipids with pyrene labelled on the fatty acid tail group (PyPC) and head group (PyPE).

This work describes the preparation and characterization of nanoscale liposomes containing lipids labelled with the excimer producing fluorophore pyrene (see Fig 1) and provides experimental evidence of excimer based liposome contrast agents for enhancement of the fluorescent signal via application of US. Advantageously this approach avoids any background signal caused by direct transmission of the excitation signal due to the large red-shift of the excimer emission with no overlap with the absorption band. It should be emphasized that this is a proof-of-principle study and despite the excitation and excimer emission wavelengths of pyrene being short in respect to tissue imaging (337 nm and 475 nm peak wavelengths respectively) the rationale for using pyrene labelled lipids is based on pyrene being a well-established and widely used fluorophore whose excimer producing behavior has been extensively studied [26]. For downstream applications in tissue imaging the emerging range of excimer producing fluorophores that can be excited and emit at longer wavelengths will need to be considered [27].

## Results

Liposomes of diameter 100 nm were produced which is a diameter that does not restrict them to future use in the circulatory system. The excimer emission intensity from liposomes exposed to US for a range of pressures used for diagnostic and therapeutic US was recorded. Liposomes containing different molar concentrations of lipids with pyrene labelled on the phospholipid fatty acid tail (1-hexadecanoyl-2-(1-pyrenedecanoyl)-sn-glycero-3-phosphocholine (β-Py-C10-HPC); abbreviated to PyPC) and also lipids with pyrene labelled on the phospholipid head group (1,2-dioleoyl-sn-glycero-3-phosphoethanolamine-N-(1-pyrenesulfonyl) (18:1 pyrene PE); abbreviated to PyPE) were studied. This methodology was chosen for identification of a suitable contrast agent formulation for the experiments of ultrasound induced fluorescence and to enable the underlying mechanisms to be investigated.

### Fluorescence spectrum measurements

**Fig 2:**
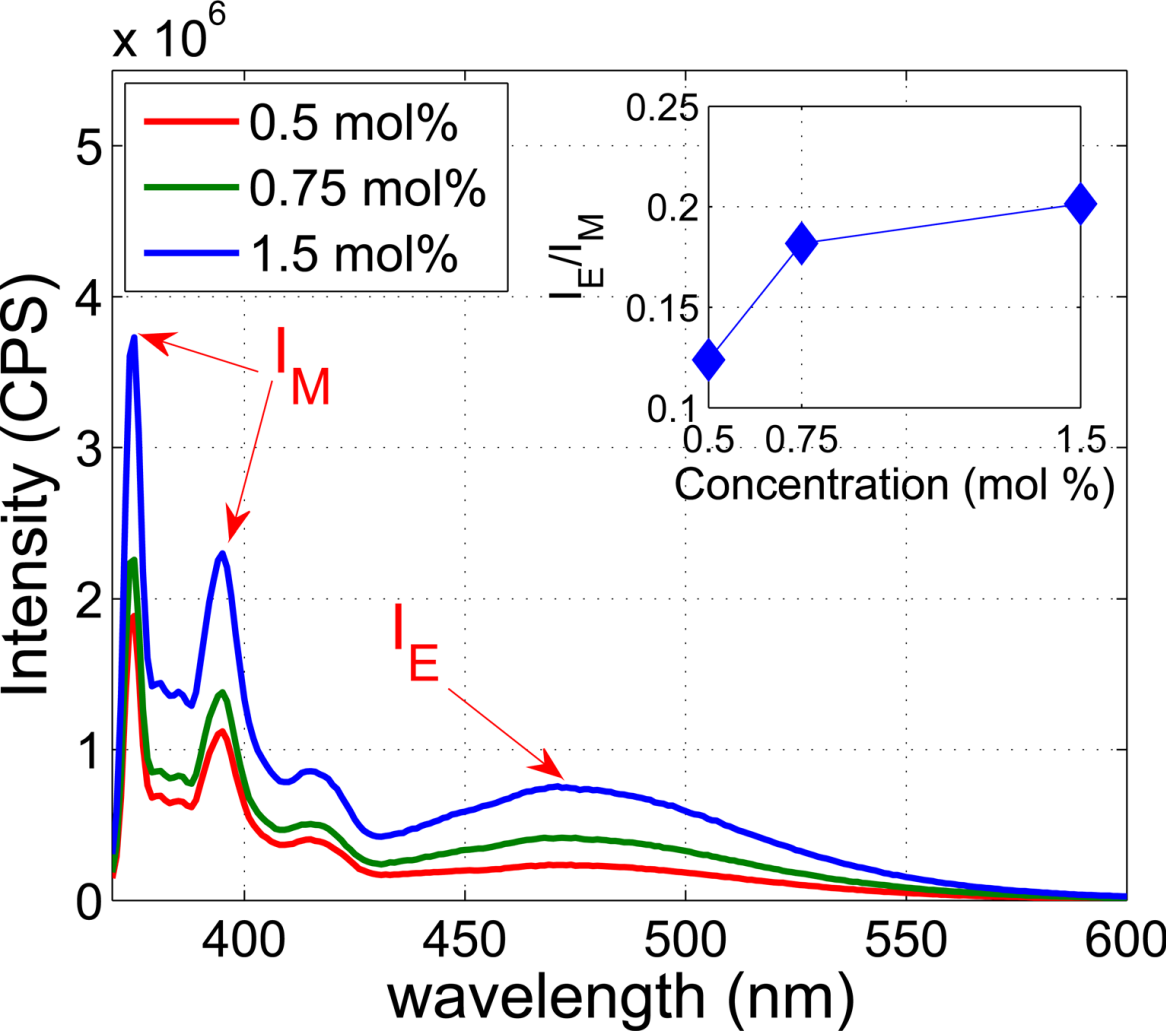
Emission spectra of liposomes containing 0.5 mol %, 0.75 mol %, and 1.5 mol % PyPC. The same total lipid (DPPC and PyPC) concentration (1.28 mM) was used in all cases. The intensity ratio of excimer (I_E_) and monomer (I_M_) for each concentration is shown inset in which data is fitted using linear interpolation. CPS: counts per second.

**Fig 3:**
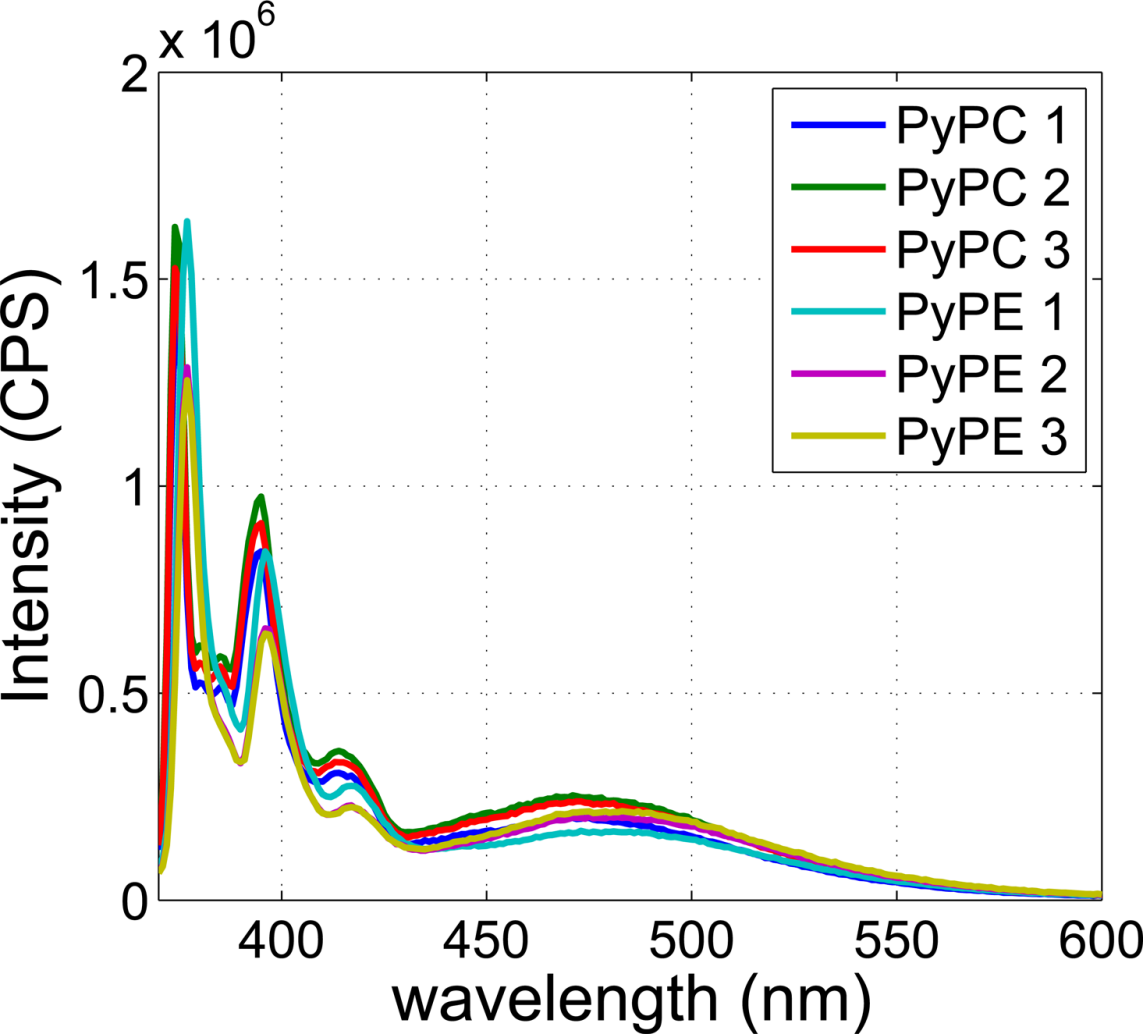
Emission spectra of liposomes containing 0.5 mol % PyPC and PyPE. Three PyPC based liposome samples (‘PyPC 1’, ‘PyPC 2’, ‘PyPC 3’) and PyPE based liposome samples (‘PyPE 1’, ‘PyPE 2’, ‘PyPE 3’) were made. The same total lipid (DPPC, PyPC and PyPE) concentration (1.28 mM) was used in all cases. CPS: counts per second.

First liposomes were prepared using a well-established freeze-thaw pressure extrusion process [28] (see Experimental Methods section) with defined phospholipids and varying amounts of pyrene. It has been observed that when the molar percentage of pyrene to phospholipid is higher than 1:120 (0.83 mol %) excimer formation occurs [29]. To minimize the background signal as well as the perturbation to the lipid bilayer, a threshold pyrene concentration was sought that was sufficient for excimer formation in the presence of US and that also displayed minimal excimer formation in the absence of US. Therefore spectra of PyPC based liposomes with PyPC concentration close to 0.83 mol % (0.5, 0.75 and 1.5 mol %) were measured to identify a suitable concentration for the US induced fluorescence experiments. Fig 2 displays the emission spectra of PyPC based liposomes for the three concentrations, which are characterized by a vibronic monomer band that peaks at a wavelength of 375 nm and a broad structure-less excimer band that peaks at a wavelength of approximately 470 nm. The extent of excimer formation is conventionally assessed by the ratio of the intensity of the excimer band at 470 nm (I_E_) and monomer band at 375 nm (I_M_) [30] and is shown inset. It can be seen from Fig 2 that I_E_/I_M_ increases with increase of concentration and the concentration of 0.5 mol % can be regarded as the threshold for excimer formation. To obtain the greatest change in fluorescence intensity upon application of US, liposomes with a 0.5 mol % concentration of pyrene labelled lipids were therefore selected and used in all subsequent experiments. A comparison was also made of the emission spectra from liposomes containing PyPC and PyPE to determine the effect the position of the pyrene moiety has on the equilibrium state excimer formation, results shown in Fig 3. Across the three batches of PyPC and PyPE labelled liposomes produced there was no clear effect due to the position of the pyrene moiety.

### Acousto-fluorescence measurements with long duration CW US

US induced excimer fluorescence was assessed by exposure of liposome solutions to CW US for a period of 14 seconds over a range of US pressures (360 kPa to 1.45 MPa) at a frequency of 2.25 MHz. The long sonication time was chosen in order to observe both a rise and fall (due to liposome rupture) of the US induced fluorescence emission. The US frequency and pressures are relevant to the typical values used in clinical diagnostic and therapeutic applications, where broadly US pressure is located in the range of 0.3–10 MPa [31] and US frequency in the range of 1–15 MHz [32].

**Fig 4:**
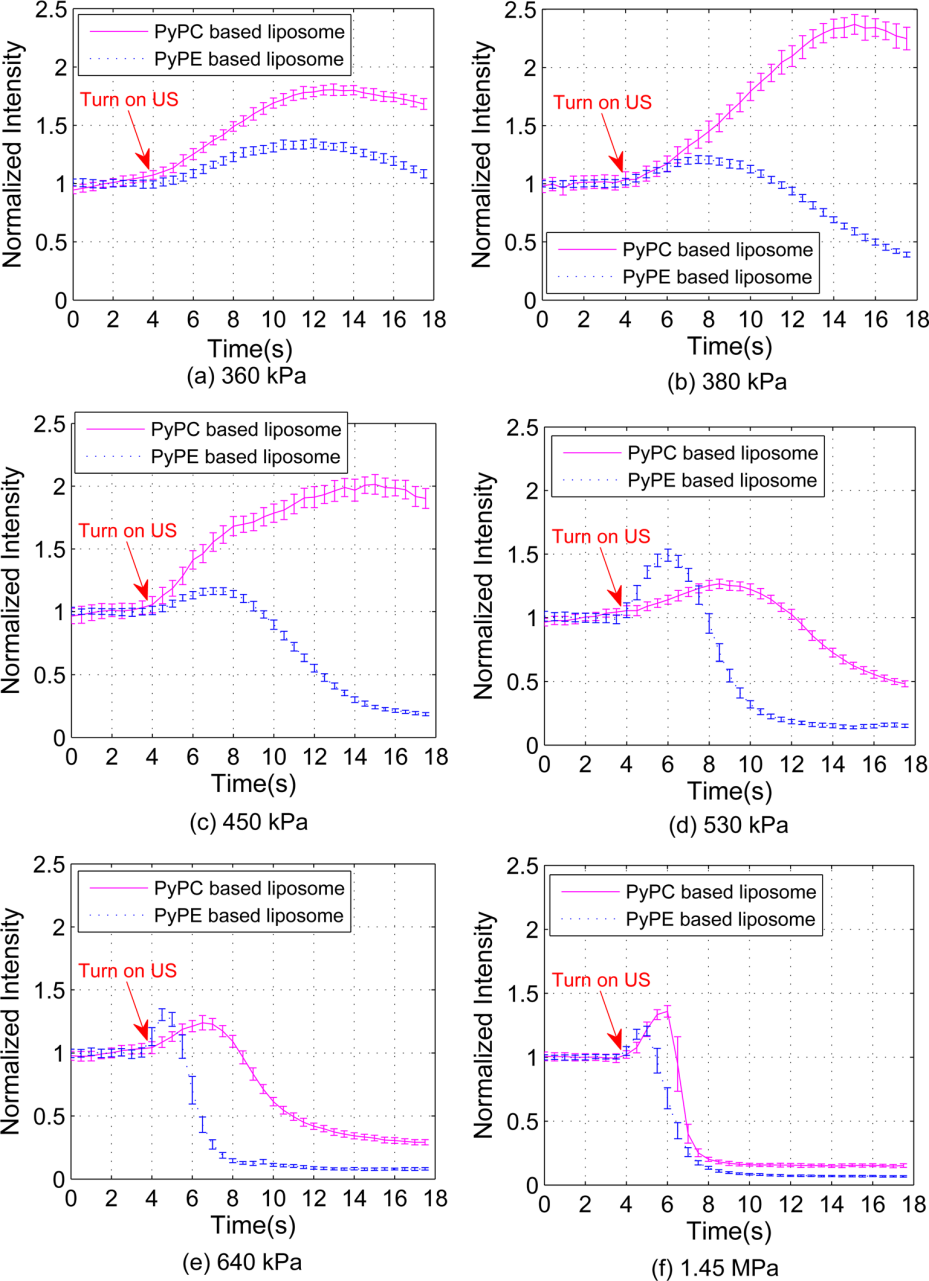
Acousto-fluorescence measurements with CW US turned on at 4 s at different US pressures (a) 360 kPa (b) 380 kPa (c) 450 kPa (d) 530 kPa (e) 640 kPa (f) 1.45 MPa for PyPC and PyPE labelled liposomes. The data were recorded continuously for 18 s with sampling period of 10 ms. 50 measurements (corresponding to 500 ms measurement time) are averaged to obtain mean and standard deviation.

Fig 4 displays the excimer intensity, normalized to the excimer emission in the absence of US, for both PyPC and PyPE labelled liposomes. Results demonstrate that US exposure causes an increase in excimer emission for all cases studied with the intensity and rise rate of emission being different for the PyPC and PyPE labelled liposomes. Of note is the maximum intensity of excimer emission and higher rate of change of fluorescence intensity from PyPC labelled liposomes at lower US pressures (360 kPa, 380 kPa, and 450 kPa) as compared to PyPE labelled liposomes. This behavior changed at higher US pressures (530 kPa, 640 kPa, and 1.45 MPa) with the PyPE labelled liposomes displaying a higher rate of excimer emission. These results demonstrate the feasibility of US induced excimer emission from the liposomes and the importance of the location of the excimer moiety in the design of liposome composition.

**Fig 5:**
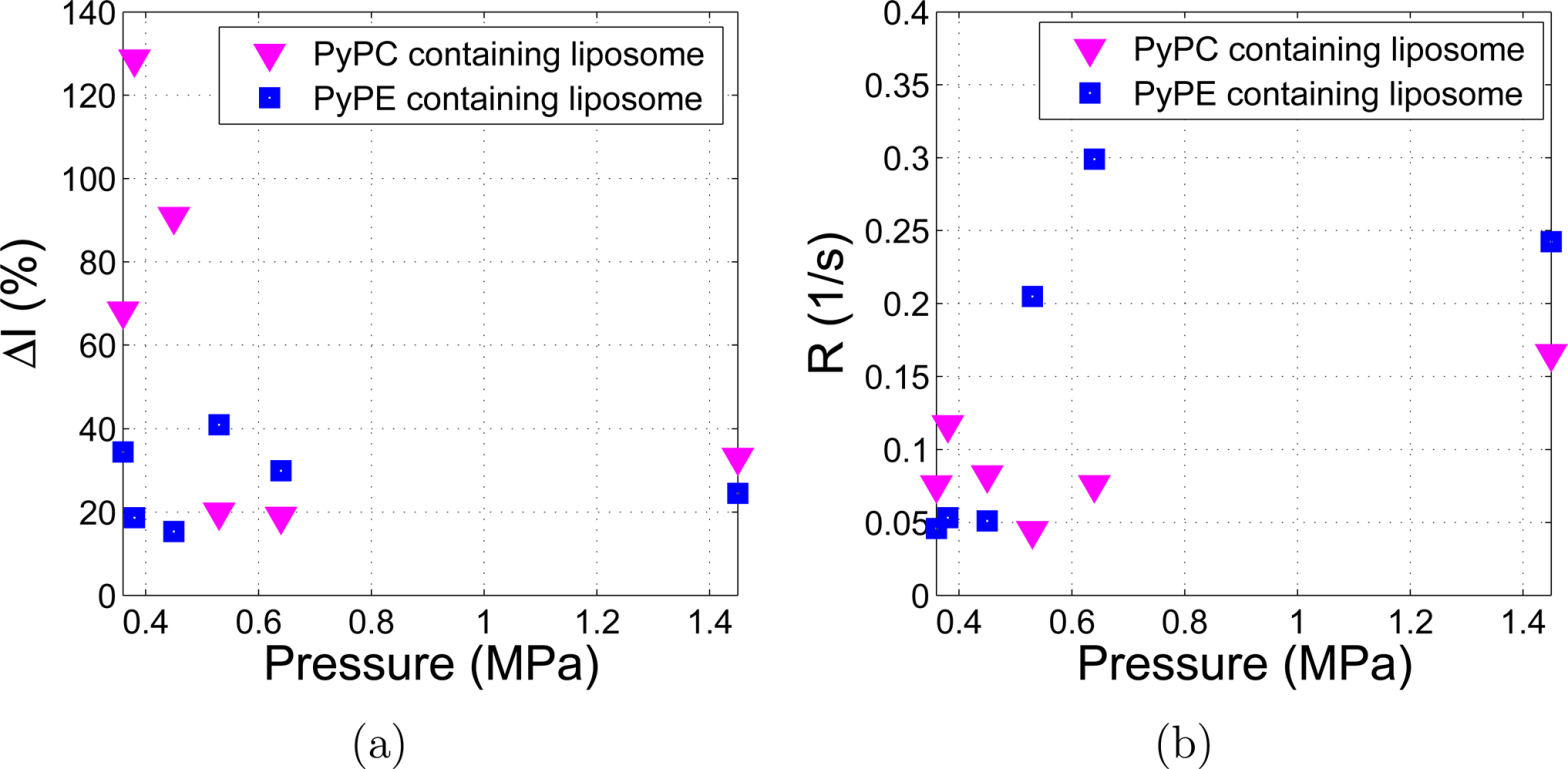
Comparison of PyPC and PyPE labelled liposomes. (a) Change in fluorescence intensity (*∆I*) against US pressure; (b) rise rate (*R*) against US pressure.

To quantify the differences in the temporal and pressure dependent variation of excimer emission intensity for the PyPC and PyPE labelled liposomes the change in fluorescence intensity (*∆I*, in %) and the rise rate of *∆I* (*R*, in s^-1^) were calculated at each pressure studied, as shown in Fig 5. Here *∆I*, shown in Fig 5A, was calculated as the ratio of the maximum increased fluorescence intensity with US on to the intensity prior to sonication while *R* was calculated by dividing the *∆I* by the sonication time interval for the emission intensity to reach the maximum intensity, shown in Fig 5B. Over the pressure range studied *∆I* of the PyPE labelled liposomes was relatively invariant ranging from approximately 20% to 40%. The PyPC labelled liposomes, however, showed a significant dependence on pressure with a variation from approximately 20% to 130% with greater *∆I* occurring at the lower end of the pressure range studied (360 kPa, 380 kPa, and 450 kPa). With reference to Fig 5B *R* of the PyPE labelled liposomes was found to vary non-linearly and significantly with pressure (ranging from 0.05 s^-1^ to 0.30 s^-1^), while it is relatively invariant for PyPC labelled liposomes (ranging from approximately 0.05 s^-1^ to 0.15 s^-1^).

### Nanoparticle Tracking Analysis

Fig 4 shows that prolonged sonication of samples leads to a reduction in excimer emission which may be due to the rupture of liposomes caused by the cavitation of gas bubbles in the surrounding media or in the bilayer itself. This effect was observed to be prevalent at high US pressures after a long period of US exposure. To investigate this further, Nanoparticle Tracking Analysis of the PyPC labelled liposomes before and after sonication (850 kPa) was carried out using Nanosight LM 14 (Malvern Instruments, Worcestershire, UK), results shown in S1A Fig. reduction of mean size (128 nm compared to 74 nm) and an increase in the liposome concentration (2.70 × 10^8^ particles/ml compared to 1.00 × 10^9^ particles/ml) after sonication were observed providing support for the cavitation hypothesis. During the cavitation process (both inertial and noninertial), larger liposomes are shattered into smaller discoid sections called bilayer phospholipid fragments which fold up into thermodynamically stable smaller liposomes [33]. Further, the early onset of PyPE liposome rupture could be associated with the relatively disordered character of the lipid bilayer as compared to the PyPC labelled liposomes.

### Acousto-fluorescence measurements with short duration CW US

**Fig 6:**
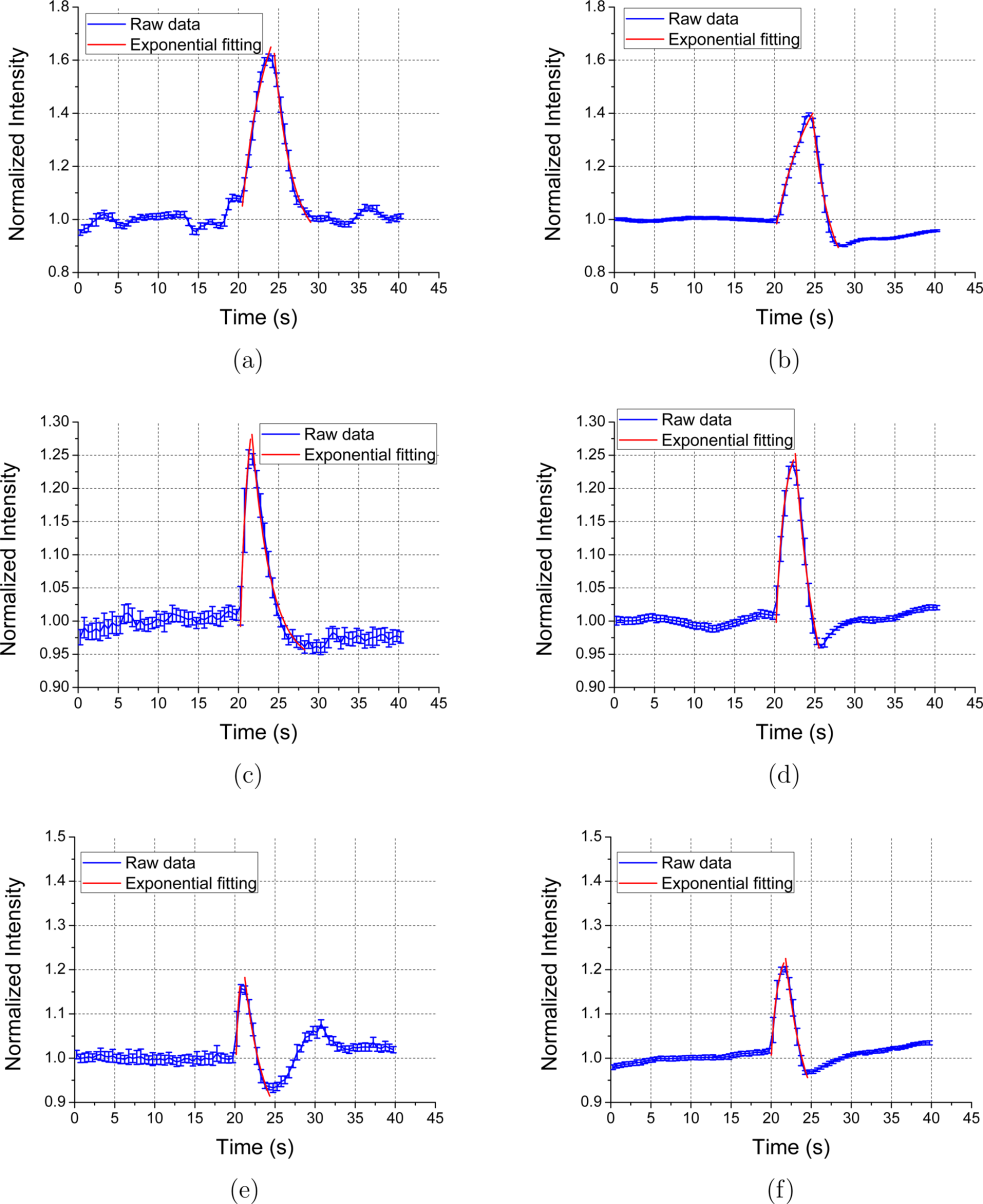
Fluorescence intensity induced by short duration US for (a) PyPC based liposomes at 430 kPa for 3.0 s; (b) PyPE based liposomes at 430 kPa for 3.0 s; (c) PyPC based liposomes at 570 kPa for 0.8 s; (d) PyPE based liposomes at 570 kPa for 0.8 s; (e) PyPC based liposomes at 850 kPa for 0.3 s; (f) PyPE based liposomes at 850 kPa for 0.3 s. The data were recorded with a sampling period of 10 ms. Three experiments were repeated with the sample replenished each time. The values from the three repeating experiments were firstly averaged and normalized by the intensity prior US sonication. The mean and standard deviation of 50 consecutive intensity values (corresponding to 500 ms measurement time) are shown. The small standard deviation (ratio of the standard deviation with the mean value is less than 4.2%) observed suggests good repeatability.

The results shown in Fig 4 demonstrate an increase in excimer fluorescence signal from pyrene labelled nanoscale liposomes following long duration CW US. To minimize the cavitation based liposome rupture induced by long duration US at high pressures as described above, the response of pyrene labelled liposomes to US with a shorter duration was studied. Here the excimer emission intensity from both PyPC and PyPE labelled liposomes at three different combinations of pressures and exposure times (430 kPa, 3 s; 570 kPa, 0.8 s; 850 kPa, 0.3 s) was studied. These exposure times were selected as they elicited the largest *∆I* at the corresponding US pressures. Results are shown in Fig 6 with the fit to the data based on a combination of an exponential rise function (*y* = *A*_*1*_exp(-*t*/*t*_*r*_)+*y*_*1*_) and an exponential decay function (*y* = *A*_*2*_exp(-*t*/*t*_*d*_)+*y*_*2*_), where *y* and *t* are the normalized intensity and time respectively, *A*_*1*_, *A*_*2*,_ *t*_*r*_, *t*_*d*_, *y*_*1*_, and *y*_*2*_ are fitting parameters. Specifically t_r_ and t_d_ are the rise and decay time constants. Table 1 summarizes the US pressure, US exposure time, fluorescence rise time constant, fluorescence decay time constant and change in fluorescence intensity obtained from Fig 6. The larger error bars for the PyPC labelled liposomes are due to lower absolute light levels detected which is likely to be caused by a batch to batch variation in the liposomes.

**Table 1:** Data obtained from Fig 6.

Liposome	Pressure (kPa)	Exposure time (s)	t_r_ (s)	t_d_ (s)	ΔI (%)
PyPC	430	3.0	2.20	2.91	50
PyPE	430	3.0	4.07	2.11	39
PyPC	570	0.8	0.88	2.38	26
PyPE	570	0.8	0.96	2.61	24
PyPC	850	0.3	0.33	2.36	15
PyPE	850	0.3	0.80	2.53	20

## Discussion

The results demonstrate that ultrasound can be used to induce an increase in fluorescence from excimer labelled liposomes. This offers the potential for a new contrast agent that can be switched on in the US focal zone and help to improve spatial resolution of fluorescence imaging. In this section possible mechanisms of US induced fluorescence from the liposomes are discussed. The movement of lipids in liposomes is known to include lipid lateral diffusion [34], “wagging” of the acyl chains [35] and transbilayer movement [36]. Each of these motions can cause a change in the fluorescence emission intensity in labelled liposomes by causing collisions between excited and unexcited molecules which forms excimers. While the results do not conclusively determine the underlying mechanism it is useful to discuss the effects of US.

Significant differences are observed in Figs 4 and 5 between the temporal and pressure dependent responses of PyPC (tail labelled) liposomes and PyPE (head labelled) liposomes. At lower US pressures (≤ 380 kPa, Fig 4A–4C), the PyPC liposomes produce a larger *ΔI* induced by US. The mechanism might relate to an increase in the “wagging” of the acyl chains upon sonication which increases the probability that two pyrene aromatic rings are brought to a planar arrangement to form excimer. This mechanism is absent in the PyPE based liposomes because the pyrene moiety is attached to the head group of the phospholipid and may explain their smaller *ΔI* at lower pressures. The large fluorescence signal increase at lower US pressures indicates the tail labelled liposomes are more likely to be useful in diagnostic US applications.

At higher US pressure (530 kPa, 640 kPa, 1.45 MPa) PyPE labelled liposomes have similar *ΔI* as PyPC based liposomes but *R* of PyPE based liposomes is significantly higher. The other known lipids motion of lateral diffusion is common to both and it is likely that the lateral diffusion is playing a role due to cavitation effects at higher US pressure. It is well known that at MPa pressure and MHz frequency US induces non-inertial cavitation during which gas bubbles pre-existing in the fluid contract and expand due to the compression and rarefaction of the medium [37]. Non-inertial cavitation may generate small cavities and create free volume in the lipid bilayer. The increased free volume can facilitate quicker lateral diffusion of the pyrene labelled lipids [38] and thus increase the collision rate of the pyrene moieties which would be observed as an increase in the excimer emission intensity for both PyPC and PyPE based liposomes. Transbilayer moment (also known as translocation or flip-flop) involves the movement of a lipid from one half of the bilayer to the other half of the bilayer. Transbilayer movement is much less likely as the rate is believed to be very slow, with half-times in the order of days in the absence of protein-mediated processes [39]. A significant increase of transbilayer movement was observed at the phase transition temperature of the lipids which is related to increased molecular packing defects [40]. However, the transbilayer movement is still of the order of several minutes [40] so it is unlikely to be the cause of the US induced fluorescence changes. In addition, acoustic radiation force (ARF) has been used to localize nanodroplets for targeted therapy [41] so one may argue that ARF might be another effect to the observed fluorescence change. For this reason the translational velocity due to ARF in our experimental system is calculated (details can be found in S1 File). With the highest US pressure applied in the experiment (1.45 MPa), the velocity is calculated as 33.5 um/s, with a direction in the US propagation direction. Based on this calculation it is feasible that a small amount of liposomes are driven into the detection field by the ARF but an equal amount can also be driven out of the detection field. Of particular note is the fact that the effect of radiation force on both the PyPC and PyPE labelled liposomes will be the same. However this does not explain the differences in the intensity of fluorescence detected for these two samples therefore ARF is also not a dominant mechanism.

Based on the above discussions a possible explanation for the US induced fluorescence is: for PyPC labelled liposomes both 1) the US increased wagging motion and 2) the increased lateral diffusion upon sonication contribute to the increased fluorescence intensity, while for PyPE labelled liposomes only the US increased lateral diffusion contributes. Since the wagging motion of a lipid is generally considered to be at a faster time scale compared with lateral diffusion [35, 42], PyPC labelled liposomes have low activation energy (the minimum energy required for excimer formation) and involve fast kinetic processes (excimer formation process). Therefore at lower US pressure the first mechanism dominates while at higher US pressure the second mechanism dominates. The higher rise rate for PyPE labelled liposomes at higher US pressure might be related to its quicker lateral mobility owing to their more disordered structure compared with PyPC labelled liposomes (due to the presence of pyrene attached to the head group). It should be noted that since excimer formation is extremely sensitive to proximity (angstrom level) and orientation of the pyrene moieties, a slight change of the lateral diffusivity of the pyrene labelled lipids or wagging motion of the pyrene attached acyl chains (for PyPC labelled liposomes) can cause variation of the excimer emission intensity.

Future work to further understand the mechanisms of the US mediated changes in fluorescence could involve comparing degassed PyPC and/or PyPE labelled liposomes with the current sample to verify the effect of cavitation. The cavitation can also be detected by analysing the acoustic radiation emanating from the solutions [33]. Future development as contrast agents will focus on the use of lipid probes containing excimers that can be excited and emit at longer wavelengths than pyrene which will allow deeper tissue to be probed. For example, the fluorophore family BODIPY, based on the 4,4-Difluoro-4-bora-3a,4a-diaza-s-indacene, are fluorescent fatty acid derivatives that have an excitation maximum at a wavelength of approximately 500 nm and form excimer emission in a concentration dependent manner at a wavelength of approximately 620 nm [27, 43, 44]. It is also worth noting that both pyrene and BODIPY exhibit relatively minor toxicological effects [45, 46]. In addition, echogenic liposomes with a lipid shell encapsulating both air and an aqueous core and several hundred nanometers in diameter are being investigated intensively as contrast agents that combine the advantages of liposomes with the high echogenicity of microbubbles [47]. Excimer-emitting echogenic liposomes could also be developed with the aim of reducing the applied US duration and pressure. This is meaningful for obtaining depth-resolved imaging along the US axis using pulsed US as in conventional US imaging. In pulsed US, the selection of US duration needs to balance the requirement for exposure times that are sufficiently long to elicit large fluorescence enhancement while avoiding rupture of the liposomes. In addition, repeatability of the agent will also need to be investigated further for effective imaging.

## Conclusions

In conclusion, a new approach to enhancement of fluorescent signals by US based on the use of nanoscale excimer labelled liposomes has been reported. An increase in excimer emission intensity following exposure to US was observed for all cases studied with the increase in intensity and time constants being different for the PyPC and PyPE labelled liposomes and dependent on the applied US pressure and exposure time. The greatest change in fluorescence intensity (130%) and fastest rise time constant (0.33 s) are achieved through the use of PyPC tail labelled liposomes. As this occurs at lower US pressures it will potentially be more useful in future applications in diagnostic US. For PyPC labelled liposomes the possible mechanism underlying the observed increase of the excimer emission intensity is suggested to arise from the ‘wagging’ of acyl chains which requires lower US pressure and has fast response. This is accompanied with the increased lipid lateral diffusivity at higher US pressures, mechanisms also active in the PyPE system.

## Experimental Methods

### Liposome preparation

Liposomes were prepared by combining appropriate concentrations of either PyPC or PyPE with unlabeled 1,2-dipalmitoyl-sn-glycero-3-phosphocholine (DPPC) phospholipids (#850355, Avanti Polar Lipids Inc, AL, USA) and formed using a freeze-thaw extrusion method [28]. This liposome preparation method was applied because it is well-established and is regarded as a reliable protocol for producing unilamellar model membrane system [48, 49]. PyPC (Life Technologies Ltd, CA, USA) or PyPE (Avanti Polar Lipids Inc, AL, USA) was reconstituted at 1 mg.ml^-1^ in methanol and chloroform in the ratio 5:1. PyPC or PyPE was mixed with DPPC phospholipids with the solvent being evaporated with oxygen-free nitrogen gas (BOC Group plc, Manchester, UK). The dried lipids were resuspended in phosphate buffered saline (PBS) buffer, to a final total lipid concentration of 12.8 mM. The buffer was made with autoclaved water. The buffered lipids underwent 5 freeze-thaw cycles using liquid nitrogen (-196°C) and water (45°C) before extruding 10 times through 100 nm track-etched membranes (Whatman Plc, Bucks, UK) using a barrel extruder (Northern Lipids Inc, Burnaby, Canada) at 45°C. The aforedescribed PBS buffer was used for all subsequent dilutions. The liposome stock solution was sealed and stored in a refrigerator at 5°C, and didn’t show visible signs of oxidation.

### Measurements of fluorescence emission spectra

The fluorescence emission spectra of the PyPC based liposomes and PyPE based liposomes in the absence of US were obtained using a spectrofluorometer (Fluoromax-4, Horiba Scientific, Kyoto, Japan). Samples were excited at a center wavelength (CWL) of 337 nm with a 2 nm bandwidth. The emission was observed between wavelengths of 370 nm and 600 nm with a 2 nm bandwidth. A quantum cuvette (105.253-QS, Hellma Analytics, Essex, UK) was used due to the ultraviolet (UV) light excitation.

### Acousto-fluorescence measurements

**Fig 7:**
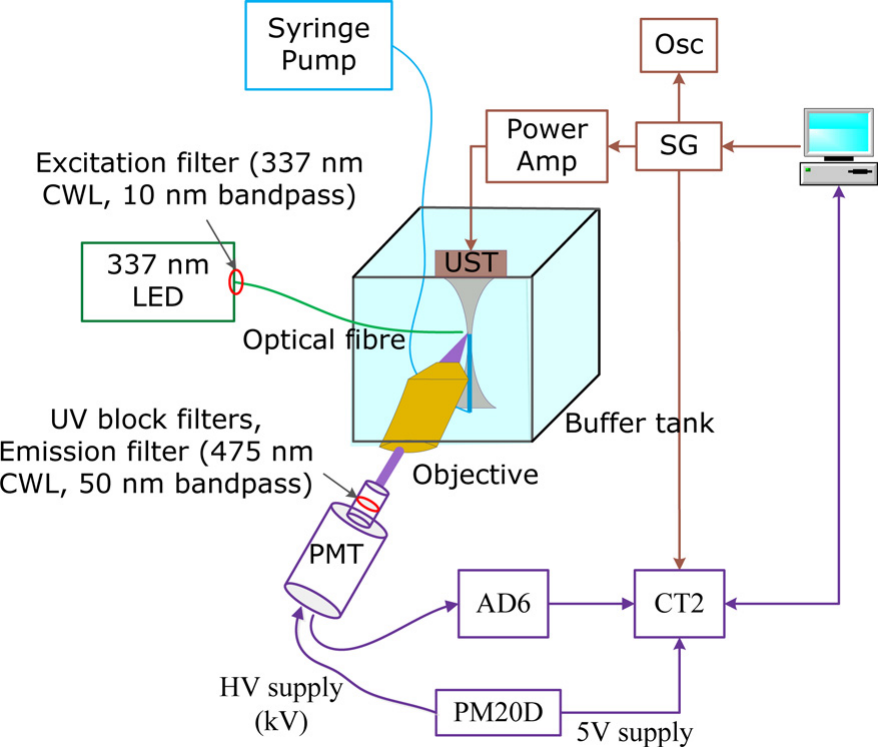
Schematic of the acousto-fluorescence experimental setup. Osc: oscilloscope; SG: signal generator; CWL: center wavelength; PMT: photomultiplier tube; AD6: amplifier-discriminator; CT2: counter/timer module; PM20D: power supply; HV: high voltage.

The experimental setup used for measurements of the US induced variation of excimer emission intensity is shown in Fig 7. The excitation light is a light emitting diode (LED) with a CWL of 337 nm (LED341W, Thorlabs, Ely, UK), coupled to a high performance bandpass excitation filter with 337 nm CWL and 10 nm full width-half max (FWHM) bandpass (#65–128, Edmund Optics, York, UK) and a 1 mm diameter optical fiber. A signal generator (AFG3022, Tektronix, Beaverton, USA) and a radio frequency power amplifier (75A250A, Amplifier Research, Souderton, USA) were employed to drive a focused 2.25 MHz US transducer (A304-SU, Olympus, Massachusetts, USA). The FWHM lateral beam width of the US transducer at focus was measured as 2.1mm using a needle hydrophone (SN1700, Precision Acoustics Ltd, Dorset, UK) with an active diameter of 0.2 mm, which was also used to measure the acoustic pressures applied in all the experiments. A syringe pump was used to inject a diluted solution of pyrene-labelled liposomes (1:10 in PBS buffer) via an open ended tube (inner diameter: 400 μm) into a buffer tank filled with PBS to maintain the osmotic pressure on the liposomes. The end of the tube was positioned in the US focal zone with the tip of the optical fiber placed in close proximity. The flow speed of the syringe pump was 0.015 ml/min. There is an initial period during which the liposome suspension gradually fills the field of view of the objective lens but no measurements were made until a constant intensity was reached.

The emitted light was collected by a long working distance objective lens (M Plan Apo 20, NA 0.42, 20×, Mitutoyo, Japan) and passed through two UV light blocking filters (UVK-2510, UQG Optics, Cambridge, UK) and a high performance emission filter with 475 nm CWL and 50 nm FWHM bandpass (#86–950, Edmund Optics, York, UK) to achieve a high rejection of the excitation, monomer emission and room light. The resulting excimer emission fluorescence was detected at DC using a photon counting system (ET Enterprises electron tubes, Uxbridge, UK) which includes the photomultiplier (PMT, 9108A), amplifier-discriminator (AD6), counter (CT2), and power supply (PM20). The signal generator (SG) and photon counting system were controlled and synchronized using Labview.

For the acousto-fluorescence measurements with long duration US, the photon counting system continuously records the excimer emission intensity with a sampling period of 10 ms. During the measurement period, the US was turned on after 4 s for 14 s. The acquired intensity was normalized according to the excimer emission intensity prior to US sonication.

For the acousto-fluorescence measurements with short duration US the US was turned on after 20 s for 3 s, 0.8 s and 0.3 s for US pressures of 430 kPa, 570 kPa, and 850 kPa respectively, and then turned off for 20 s while continuously detecting light. This procedure was repeated three times after replenishing the sample using the syringe pump. The averaged values of the three measurements were firstly calculated and normalized by the intensity prior US sonication. 50 consecutive intensity values corresponding to 500 ms measurement time were then averaged and the mean and standard deviation were plotted for each acousto-fluorescence measurement.

## Supporting Information

S1 Fig Size distribution of PyPC labelled liposomes. (DOCX)

S1 File Calculation of acoustic radiation force. (DOCX)
