# Supplementary material for: Ultrasound Induced Fluorescence of Nanoscale Liposome Contrast Agents
Source: PLoS One. 2016 Jul 28;11(7):e0159742. doi: 10.1371/journal.pone.0159742 (PMC4965150; doi:10.1371/journal.pone.0159742)
Supplement: S1 Fig — (DOCX) [file pone.0159742.s001.docx]

# Size distribution of PyPC labelled liposomes

Fig S1. Size distribution of PyPC labelled liposomes before and after US exposure (850 kPa pressure amplitude). Measured using Nanosight LM14.
