# Supplementary material for: Ultrasound Induced Fluorescence of Nanoscale Liposome Contrast Agents
Source: PLoS One. 2016 Jul 28;11(7):e0159742. doi: 10.1371/journal.pone.0159742 (PMC4965150; doi:10.1371/journal.pone.0159742)
Supplement: S1 File — (DOCX) [file pone.0159742.s002.docx]

# Calculation of acoustic radiation force

The acoustic radiation force to the liposome in our experimental system can be calculated using the following equation [1]:

$$F_{\mathrm{rad}}=\frac{1}{3}\pi\rho A^{2}{(kR_{0})}^{3}{(\frac{\delta_{v}}{R_{0}})}^{2}[\frac{ϒ-1}{\lambda_{\alpha}}-\frac{\tilde{ϒ}\lambda_{\rho}c^{2}}{\tilde{c}^{2}}-2-4Re(\Lambda)]$$

where

$$\Lambda=\frac{3+16\lambda_{\eta}-\frac{19}{\lambda_{\eta}}+4\sigma\left( k_{v}R_{0} \right)^{2}(5+2\lambda_{\eta})/\rho w^{2}R_{0}^{3}}{89+48\lambda_{\eta}+\frac{38}{\lambda_{\eta}}-40\sigma\left( k_{v}R_{0} \right)^{2}(1+\lambda_{\eta})/\rho w^{2}R_{0}^{3}}$$

$\delta_{v}=\sqrt{2\eta/\rho w}$, $A=\frac{P_{a}}{\rho w}$, $k=\frac{w}{c}$, $\lambda_{\alpha}=\frac{\alpha}{\alpha_{s}}$, $\lambda_{\rho}=\frac{\rho}{\rho_{s}}$, $\lambda_{\eta}=\frac{\eta}{\eta_{s}}$,$w=2\pi f$, $k_{v}=(1+i)/\delta_{v}$.

The other parameters are summarized in Table 1. Typical values from literature are used for the calculation.

S2 Table List of Symbols

| Symbol | Meaning | Value |
| --- | --- | --- |
| $\rho$ | fluid density | 1000 kg/m3 |
| $P_{a}$ | pressure amplitude | 1.45 MPa |
| f | ultrasound frequency | 2.25 MHz |
| c | ultrasound speed in the fluid | 1500 m/s |
| η | dynamic viscosity of the fluid | 0.001 Pa·s [2] |
| $R_{0}$ | radius of the liposome | 50 nm |
| $ϒ$ | ratio of specific heat in the fluid | 1.33 [3] |
| $\alpha$ | volume thermal expansion coefficient of the fluid | 0.257 × 103 1/K [4] |
| $\alpha_{s}$ | volume thermal expansion coefficient of DPPC | 0.00101 1/K [5] |
| $\rho_{s}$ | density of DPPC | 800 kg/m^3^ [6] |
| $\eta_{s}$ | dynamic viscosity of DPPC | 0.08 Pa·s [2] |
| $\sigma$ | surface tension | 30 × 10-3 N/m [7] |

The speed due to the acoustic radiation force ($u_{rad}$) can be further calculated by [8]:

$$u_{\mathrm{rad}}=\frac{(\eta+\tilde{\eta})F_{\mathrm{rad}}}{2\pi(2\eta+3\tilde{\eta})\eta R_{0}}$$

Note that tilde over variable denotes quantities that concern the medium inside the liposome. For a liposome, because the medium inside and outside of the lipid bilayer are the same, $\tilde{ϒ}=ϒ$, $\tilde{c}=c$, $\tilde{\eta}=\eta$. For the highest ultrasound pressure applied in the experiment ($P_{a}$ = 1.45 MPa), the calculated $u_{\mathrm{rad}}$ is 33.5 µm/s.

1. Doinikov AA. Acoustic radiation forces: Classical theory and recent advances. Recent Res. Devel. Acoustics. 2003; 1(2003): 39-67.

2. Hill RJ, Wang CY. Diffusion in phospholipid bilayer membranes: dual-leaflet dynamics and the roles of tracer-leaflet and inter-leaflet coupling. Proc. R. Soc. A Math. Phys. Eng. Sci. 2014; 470(2167).

3. Salby ML. Physics of the Atmosphere and Climate. Cambridge University Press, 2012.

4. Irvine TF, Duignan MR. Isobaric thermal expansion coefficients for water over large temperature and pressure ranges. Int. Commun. Heat. Mass. 1985; 12(4): 465-478.

5. Marsh D. CRC handbook of lipid bilayers. CRC Press, 1990.

6. Buehler L. Cell Membranes. Taylor & Francis Group, 2015.

7. Vicario-de-la-Torre M, Benitez-del-Castillo JM, Vico E, Guzman M, De-Las-Heras B, et al. Design and Characterization of an Ocular Topical Liposomal Preparation to Replenish the Lipids of the Tear FilmOcular Topical Liposomal Preparation. Invest. Ophthalmol. Vis. 2014; 55(12): 7839-7847.

8. Dayton PA, Zhao S, Bloch S, Schumann P, Penrose K, et al. Application of Ultrasound to Selectively Localize Nanodroplets for Targeted Imaging and Therapy. Mol. Imaging. 2006; 5(3): 160-174.
